# Intestinal Parasitic Infection: Prevalence and Associated Risk Factors at Delgi Primary Hospital, Northwest Ethiopia

**DOI:** 10.1155/tswj/8787678

**Published:** 2025-03-21

**Authors:** Tarekegn Addis, Tilahun Yohannes

**Affiliations:** Department of Biology, College of Natural and Computational Sciences, University of Gondar, Gondar, Ethiopia

**Keywords:** Delgi Primary Hospital, intestinal parasites, prevalence, risk factors

## Abstract

Intestinal parasitic infections (IPIs) are among the leading causes of mortality and morbidity in developing nations such as Ethiopia. Determining epidemiological information of IPI is crucial for effective public health planning and intervention. The present study is aimed at assessing the prevalence and associated risk factors of human IPIs at Delgi Primary Hospital, Central Gondar Zone, Northwest Ethiopia. An institutional-based cross-sectional study was conducted from March to May 2023. A stool specimen was collected from 404 selected participants and examined microscopically for the presence of developmental stages of the intestinal parasites. A structured questionnaire was used to obtain information regarding the sociodemographic and associated risk factors. Data were analyzed using SPSS Version 23, and a crude odd ratio was calculated to verify and measure the possible association between IPIs and potential risk factors. A *p* value < 0.05 was taken as statistically significant. The overall prevalence of IPIs was 47.20%. Six species of intestinal parasites were identified: *Entamoeba histolytica/dispar* (16.8%) was the most predominant parasite, followed by *Giardia lamblia* (9.9%), *Ascaris lumbricoides* (8.1%), Hookworm species (7.6%), *Schistosoma mansoni* (4.2%), and *Hymenolepis nana* (0.5%). Furthermore, double and triple parasitic infections were observed in 10.39% and 0.49% of the study participants, respectively. Not having a habit of handwashing after toilet (AOR = 2.048, CI = 0.694, 3.583, *p* = 0.001), the habit of eating unwashed vegetables (*AOR* = 3.046, *CI* = 0.685, 5.596, *p* = 0.016), and presence of dirt matter under the nail (*AOR* = 2.939, *CI* = 0.621, 4.418, *p* = 0.001) were found to be significantly associated risk factors. This study showed that IPIs remained a public health concern in the study area. Therefore, regular provision of health education on personal hygiene and sanitation is recommended to prevent and control IPIs in the study area.

## 1. Background

One of the most significant and serious public health problems in developing countries, such as Ethiopia, is intestinal parasitic infection (IPI) [1]. The IPIs are the main cause of morbidity and occasional mortality among the infected population, especially in tropical and subtropical regions around the world [2]; the majority of these cases are children, which are linked to their poor hygienic practices and weak immune status [3]. Intestinal parasites can be categorized into two main groups: protozoan and helminths. The major intestinal parasites are the protozoan species, such as *E. histolytica* and *G. intestinalis*, and helminths species like *A. lumbricoides*, *Trichuris trichiura*, hookworm, *H. nana*, and Schistosomiasis [1].

Intestinal protozoan parasites have a simple life cycle with cyst and trophozoite life stages. The cyst is a resistant stage excreted with the stool of an infected person and then infects a new host due to ingestion with contamination via the fecal–oral route [4]. After ingestion, the cyst wall is dissolved, and the organism excysts within the lumen of the small intestine and then gives rise to trophozoites, the active stage. The trophozoite stage keeps reproducing and feeding and then causes the disease [3]. Parasitic helminths are multicellular worms with egg, larvae, and adult life stages. Helminths are mainly transmitted by the ingestion of infective eggs by means of the fecal–oral route and intact skin penetration by the infective stage larvae of soil-transmitted helminths [5].

The IPIs are more prevalent among the poor segment of the population and closely associated with limited access to clean water, poor hygiene and sanitation, unsafe human waste disposal and open field defecation, inadequate health services, and a low level of awareness [6]. In addition to causing high levels of morbidity and mortality, IPIs are responsible for nutritional deficiency, including iron deficiency, vitamin deficiency, protein energy malnutrition, seizures, portal hypertension, chronic diarrhea [7], impaired physical and mental development in children, decreased body weight in pregnancy, and low birth weight [3]. According to WHO guidelines [8], IPIS can be easily prevented and controlled through proper keeping of hygiene and sanitation, proper treatment and waste disposal, maintaining clean potable water, consuming properly cooked and washed foods, avoiding unsafe contact with soil and infected water bodies, wearing shoes, and a periodic deworming program for children in high-risk areas.

Like in other developing countries, IPIs are responsible for extensive morbidity and mortality in Ethiopia. According to the report from the Ministry of Health, IPIs are the second most common cause of outpatient morbidity next to malaria in Ethiopia [9]. Although studies have been conducted on the distribution and prevalence of intestinal parasites in Ethiopia, there are still several localities for which epidemiological information is not available [10]. Information obtained from Delgi Primary Hospital indicated that a significant proportion of the community is affected by IPIs. However, the public health burden of IPIs has not been studied in the current study area. Therefore, this study was designed to determine the prevalence and associated risk factors of human IPIs at Delgi Primary Hospital, Central Gondar Zone, Northwest Ethiopia.

## 2. Materials and Methods

### 2.1. Study Area Description

The study was conducted at Delgi Primary Hospital in Takussa District, Central Gondar Zone, and Northwest Ethiopia. The district is located about 831 km Northwest of Addis Ababa and about 104 km southwest of Gondar town. This town has a latitude and longitude of 11°5⁣′30⁣^″^ E 12°23⁣′30⁣^″^ E, coordinates of 35°58⁣′0⁣^″^E 37°53⁣′30⁣^″^ E, and an elevation of 1785 m above sea level. The minimum and maximum annual temperatures are 15°C and 24°C during the rainy season, respectively. Daily temperatures become high during the months of March to May, where it rises as high as 37°C. Nearly all of the land in the district is in the lowlands. The mean annual rainfall for the area is about 870 mm. The rainy months extend from June until the end of October.

### 2.2. Study Design and Period

An institutional-based cross-sectional study was conducted to assess the prevalence and associated risk factors of human IPIs among individuals attending Delgi Primary Hospital from March to May 2023.

### 2.3. Study Population

The study population was all patients showing symptoms of parasitic infection and requested for stool examination at Delgi Primary Hospital, Central Gondar Zone, during the study period.

### 2.4. Inclusion and Exclusion Criteria

The study has included those who visited the hospital during the data collection period, signed the informed consent voluntarily, and did not take any antiparasitic drugs within the last 3 months. However, those who have no IPI symptoms, did not voluntarily participate, and took antiparasitic drugs in the last 3 months were not included.

### 2.5. Study Variables

#### 2.5.1. Dependent Variable

The dependent variable is the prevalence of intestinal parasites.

#### 2.5.2. Independent Variables

The independent variables are both sociodemographic characteristics and associated factors of IPIs.

### 2.6. Sample Size Determination and Sampling Technique

#### 2.6.1. Sample Size Determination

The sample size (the required *n*) was determined by using the single population proportion formula as it was implemented in Naing et al. [11].

 $$\begin{array}{c} N=\frac{Z2*p\;\left(1-p\right)}{D2} \end{array}$$

As no comparable research has been conducted in this area previously, a 50% prevalence rate of IPIs was taken. Therefore, the required number of sample size (*N*) was calculated to be 384. To account for potential nonresponse and ensure adequate statistical power, a 5% contingency (approximately 20) was added to the calculated sample size, resulting in a final sample of 404 participants.

#### 2.6.2. Sampling Technique

A systematic probability sampling technique was employed and every third patient who arrived at the hospital during the study period was asked to participate until the desired sample size was reached.

### 2.7. Methods of Data Collection

#### 2.7.1. Data Collection Through Questionnaires

A structured questionnaire was developed in English and then translated into the local language, that is, Amharic. Sociodemographic characteristics and associated factors were collected using this structured questionnaire. The questionnaire was pilot tested and revised before being used for real data collection.

#### 2.7.2. Collection of Stool Samples and Parasitological Examination

Fresh stool samples were collected from each participant. Participants were instructed to bring a small portion (approximately 5 g) of stool in a clean, labeled collection cup. Applicator sticks were provided. The date of sampling, participant code, age, and sex were recorded for each participant. After examining the specimens for consistency, color, the presence of blood, mucus, and adult intestinal helminths, macroscopically, a direct wet mount and then Formal-ether concentration slides were prepared and examined according to the WHO guideline [12]. The slide preparation and examination were carried out by experienced laboratory technologists working at the Delgi Primary Hospital.

#### 2.7.3. Data Quality Control Technique

A structured questionnaire was developed in English and translated into Amharic (local language) and then back-translated into English to check for any inconsistencies or distortions in the meaning of words and concepts. The questionnaire was pretested on other 10% of patients before the actual data collection date in the area. To ensure the quality control, the entire laboratory procedures including the collection and handling of specimens were carried out with standard protocol, and disposable gloves were worn. For quality assurance, instruments and reagents were checked for reliability and reproducibility of the test before any test was started. The collected data were registered in the appropriate format.

### 2.8. Ethical Consideration

The official permission letter for ethical clearance was obtained from the College of Natural Computational Sciences Research Ethics Review Committee, University of Gondar (Reference No. CNCS/34/02/203/2023). Further permission was obtained from Delgi Primary Hospital and laboratory staff. Prior to data collection, all participants were provided with detailed information about the study's objectives. Informed consent was obtained from adult participants, while written consent from parents or legal guardians was secured for participants under the age of 18. All data collected in the study were kept confidential. IPI-positive individuals were treated with proper drugs by the physician of the hospital.

### 2.9. Data Analysis

All data was entered and analyzed using SPSS Version 23, and results were expressed using frequency and percentages. Figures and tables were used for data presentation. Logistic regression (both univariate and then multivariate) was used to assess the strength of association and the effect of independent variables on the prevalence of IPI. Univariate analysis was performed first, and variables with *p* value < 0.25 were transferred to multivariate analysis. In all cases, a 95% confidence interval was used, and *p* < 0.05 was considered statistically significant.

## 3. Results

### 3.1. Sociodemographic Characteristics of the Study Participants

Of the 404 participants, 210 (52%) were male, and 194 (48%) were female. Regarding age, 151 (37.4%) were 5–14 years old, 151 (37.4%) were 15–24 years old, and 102 (25.2%) were 25 or older. In terms of education, 74 (18.3%) were illiterate, 138 (34.2%) had primary education, 122 (30.2%) had secondary education, and 70 (17.4%) had a diploma or higher (Table 1).

### 3.2. Prevalence of IPIs

Based on the result of the microscopic examination of stool specimens, a total of 6 different species (2 protozoans and 4 helminths) of intestinal parasites were identified. The overall prevalence of IPI was 47.20% (191/404). *E. histolytica/dispar* (16.83%, 68/404) was the dominant parasite followed by *G. lamblia* (9.90%, 40/404), *A. lumbricoides* (8.17%, 33/404), hookworm species (7.67%, 31/404*), S. mansoni* (4.21%, 17/404), and *H. nana* (0.50%, 2/404). Furthermore, double and triple parasitic infections were observed. The number of double infections was 41, out of these, 8 (19.51), 8 (19.51), 7(17.07), and 7 (17.07) were infections of *E. histolytica/dispar* and *S.* mansoni, *A. lumbricoides* and hookworm, *E. histolytica/dispar* and *A. lumbricoides*, and *G. lamblia* and hookworm, respectively. The overall intestinal parasite burden among females was 96/191 (50.26%) and that of males was 95/191 (49.74%) (Table 2).

### 3.3. Analysis of Risk Factors Associated With IPI

#### 3.3.1. Bivariate Logistic Regression Analysis of Sociodemographic Factors and IPIs

In the univariate analysis of the sociodemographic characteristics of the study participants, study subjects within the age group from 5 to 14 years were about three times more likely to be infected with IPIs than the age group above 25 (COR = 2.838, CI = 0.499–4.409, *p* = 0.005). Regarding income level, study subjects who had 1000–3000 ETB monthly income were more than two times more infected with IPIs than those who had > 3000 ETB monthly income (COR = 2.672, CI = 0.593–4.593, *p* = 0.014) (Table 3).

### 3.4. Lifestyle-Related Factors

As indicated in Table 4, lifestyle-related factors like the habit of consuming unwashed vegetables and handwashing before eating food had statistically significant associations with IPIs. However, lifestyle-related factors such as the habit of shoe wearing and eating raw meat were not significantly associated with the prevalence of IPIs among study subjects. Individuals who consumed unwashed vegetables were three times more likely to contract IPIs compared to those who did not (COR = 3.046, CI = 0.685–5.596, *p* = 0.016). Similarly, study subjects who washed their hands before eating food were about three times more likely to be infected with IPIs than those who did not wash their hands before eating food (COR = 2.866, CI = 0.576–3.302, *p* = 0.005).

### 3.5. Personal Hygiene Practices

Personal hygiene practices, including the presence of dirt under fingernails, access to a latrine, handwashing habits, and the frequency of handwashing after using the toilet, were significantly associated with IPIs (Table 5). Study subjects who had dirt matter under their fingernails were about two times more likely to be infected with IPIs than those who did not have dirt matter under their fingernails (COR = 2.939, CI = 0.621, 4.418, *p* = 0.001); study subjects who lacked access to home latrines had a threefold increased risk of infection with IPIs than those who had a latrine at home (COR = 3.78CI = 0.518, 5.177, *p* = 0.005). Similarly, those study subjects who did not wash their hands after using the toilet were one time more likely to acquire IPIs than those who washed their hands after using the toilet (COR = 1.075, CI = 0.628, 1.840, *p* = 0.012); (Table 5).

#### 3.5.1. Multivariate Logistic Regression Analysis of Selected Variables

All sociodemographic, hygiene, and lifestyle variables with a *p* value less than 0.25 in the bivariate logistic regression analysis were included in the multivariate logistic regression model. Among the potential risk factors, handwashing before eating food, age group, income level, habit of handwashing after using the toilet, habit of eating unwashed vegetables, presence of latrine at home, and presence of dirt matter under fingernails were significantly associated with IPIs (*p* < 0.05). Study subjects in the age group 5–14 were nearly times more likely to be infected with IPIs than study subjects in the age group above 25 (AOR = 1.835, CI = 0.491–2.419, *p* = 0.001). Individuals who neglected to wash their hands after using the toilet were three times more likely to contract IPIs compared to those who practiced proper hand hygiene (AOR = 3.941, CI = 0.619–5.432, *p* = 0.001). With regard to the feeding habits, study subjects who ate unwashed vegetables were two times more likely to acquire IPIs than those who did not eat unwashed vegetables (AOR = 2.987, CI = 0.642–4.517, *p* = 0.013). Similarly, Those participants whose fingernails contained dirt materials were three times more likely to be infected with IPIs than those who did not have dirt materials in their fingernails (AOR = 3.934, CI = 0.615–5.417, *p* = 0.001) (Table 6).

## 4. Discussion

Determining the prevalence and risk factors of IPIs is crucial for developing effective prevention and control strategies. In line with this view, this study is aimed at assessing these factors among individuals at Delgi Primary Hospital. The overall prevalence of IPIs among these study subjects was 47.28%. This prevalence was comparable with the findings of the studies conducted in Yabelo General Hospital, Ethiopia, which was 48% [13]. It is higher than the result of Jimma Health Center, Ethiopia (20.6%) [9]. However, it is lower than the result from Sanja Primary Hospital, Ethiopia (52.9%) [14]; Teaching hospitals in Zagazig District, Egypt (56%) [15]; Shahura Health Center, Ethiopia (56.9%) [4]; Teda Health Centre, Ethiopia (62.3%) [16]; and University Hospital of Bobo-Dioulasso, Burkina Faso (65.3%) [6]. These variations may be due to differences in conducting the survey time, living conditions of the study participants, level of environmental sanitation, drinking water source, and geographical factors in the study areas. The relatively higher rates of IPIs among study subjects at Delgi Primary Hospital compared to the mentioned studies may be due to the habit of eating unwashed vegetables, lack of latrine per home, and poor habit of handwashing after using the toilet.

In the present study, *E. histolytica/dispar* (16.8%) was the most predominant IPI. This prevalence was in agreement with the reports by Tigabu et al. [4] in Shahura Health Center, Northwest Ethiopia. In both findings, the most significantly associated risk factors for the occurrence of *E. histolytica/dispar* were handwashing habits, eating unwashed vegetables, and dirt matter under fingernails. Furthermore, this parasite was predominant in the results of Sanja Primary Hospital, Ethiopia (19.8%) [14] and Yabelo General Hospital, Ethiopia (22.6%) [13]. On the other hand, it was second and third level in research outcomes from Teaching hospitals in Zagazig district, Egypt (10%) [15], and Teda Health Centre, Ethiopia (4.6%) [16], respectively. The possible reasons for the higher dominance of Amoebiasis might be due to the level of contamination of potable water, poor handwashing habits after using the toilet, contamination of food, and eating food without washing hands.

The second most prevalent parasite in the present study was *G. lamblia* (9.9%). The prevalence rate of the present study was much lower than the prevalence observed in Teda Health Center, Ethiopia (12.4%) [16]; Yabelo General Hospital, Ethiopia (15.3%) [13]; and Bereka Medical Center, Ethiopia (23.7%) [17]. However, the result of giardiasis in this study was higher than the findings from Shahura Health Center, Ethiopia (5.5%) [4], and the University Hospital of Bobo-Dioulasso, Burkina Faso (4.8%) [6]. According to the multivariate analysis in this study, handwashing before food, the habit of handwashing after the toilet, the habit of eating unwashed vegetables, and dirty matter under the nails were among the determinant risk factors. Therefore, this result supports the distribution of giardiasis, as all of the aforementioned factors are the possible routes of transmission of the parasite.

The third most prevalent parasite in this study was *A. lumbricoides* (8.17%). Its prevalence was much lower than the prevalence rate reported in Teda Health Center, Ethiopia (23.2%) [16]. The result is comparable with the results from Teaching hospitals in Zagazig District, Egypt (8.8%) [15] and Jimma Health Center, Ethiopia (5.7%) [9] but much higher than IPI prevalence study outcomes from University Hospital of Bobo-Dioulasso, Burkina Faso (0.3%) [6]; Shahura Health Center, Ethiopia (1.1%) [4]; Yabelo General Hospital, Ethiopia (1.8%) [13]; and Sanja Primary Hospital, Ethiopia (2.1%) [14]. The *A. lumbricoides* is the largest roundworm that infects humans and is the most common helminthic infection worldwide, mainly in the tropical and subtropical regions of the world where there is poor sanitation and local soil is contaminated with human feces [18]. Ethiopia has one of the highest rates of Ascariasis prevalence in Africa; one-third of the population is estimated to be infected with *A. lumbricoides* [19]. As is well known, the distribution and transmission of *A. lumbricoides* are mainly attributed to poor personal hygiene, soil contamination with infected human and animal faeces, and handwashing practice before meals and after the toilet.

Double and triple parasitic infections were also observed in the present study. Of the total infections, 10.39% of individuals had double infections and 0.49% of individuals had triple infections, which were lower than the findings of the study conducted in Bereka Medical Center southeast Ethiopia (5.6%) [17]. The prevalence of mixed infection is mainly due to lower awareness about IPIs, poor personal hygiene, favorable environmental conditions, vulnerable human behavior, and socioeconomic factors.

The current study also assessed the potential association of IPIs with the possible risk factors. Even though there were many factors associated with intestinal parasites, the logistic regression analysis in the present study indicated that some of them showed a statistically significant association with IPIs. The determinant factors of IPIs in the study subjects were handwashing before eating food, age group, income level, habit of handwashing after using the toilet, habit of eating unwashed vegetables, presence of a latrine at home, and presence of dirt matter under fingernails. The multivariate analysis confirmed that individuals who did not wash their hands after defecation were approximately four times more likely to contract IPIs compared to those who did (AOR = 3.941, CI = 0.619–1.432, *p* = 0.001). This finding aligns with a previous study conducted in Teda Health Center, Northwestern Ethiopia [16]. Inadequate hand hygiene can lead to fecal-oral contamination, increasing the risk of infection.

Regarding the feeding habit, participants who consumed unwashed vegetables were nearly three times more likely to contract IPIs than those who did not (AOR = 2.987, CI = 0.642–4.517, *p* = 0.013). This might be due to contaminated vegetables being a source of parasite infection if they are consumed without proper washing or cooking. The other determinant factor was dirty matter under nails; those participants whose fingernails contained dirt materials were more than three times more likely to be infected with IPIs than those who did not have dirt materials in their fingernails (AOR = 3.934, CI = 0.615–1.417, *p* = 0.001). This could be attributed to the potential contamination of their fingers with soil containing cysts and eggs of parasitic organisms, which can lead to intestinal infections. The proportions of females with IPIs (38.1%) were higher than that of males (34.4%). The higher prevalence in females might be due to females engaging more in food preparation, taking care of family hygiene, and home and environmental cleaning tasks.

## 5. Conclusions

The overall prevalence of IPI among study subjects attending Delgi Primary Hospital was 47.2%. The proportions of infection were higher for protozoa compared to helminths. *E. histolytica/dispar* among the protozoans and *A. lumbricoides* among the helminths were the predominant intestinal parasites. Furthermore, double and triple parasitic infections were observed. According to the multivariate logistic regression analysis of this study, age group, the habit of handwashing after using the toilet, the habit of eating unwashed vegetables, dirt matter under the nails, and the presence of a latrine at home were the predictor factors associated with the occurrence of IPIs. Therefore, the community should receive health education regarding personal hygiene and sanitation, avoiding eating unwashed vegetables and raw meat, and proper nail trimming.

## Figures and Tables

**Table 1:** Sociodemographic characteristics of study participants, Delgi Primary Hospital, Central Gondar, Ethiopia (*n* = 404).

**Characteristics**	**Categories**	**Frequency (** **n** = 404**)**	**Percentage (%)**
Sex	Male	210	52.0
	Female	194	48.0

Age group (years)	5–14	151	37.4
	15–24	151	37.4
	Above 25	102	25.2

Residence	Urban	158	39.1
	Rural	246	60.9

Marital status	Single	170	42.1
	Married	115	28.5
	Divorced	76	18.8
	Windowed	43	10.6

Education level	Illiterate	74	18.3
	Primary school	138	34.2
	Secondary school	122	30.2
	Diploma and above	70	17.3

Occupational status	Governmental employee	58	14.4
	Merchant	62	15.3
	Farmer	50	12.4
	Housewives	15	3.7
	Student	219	54.2

Family size	< 3	83	20.5
	4–6	127	31.4
	7–9	117	29.0
	> 9	77	19.1

Income level (in ETB)	< 1000	128	31.7
	1000–3000	157	38.9
	> 3000	119	29.5

**Table 2:** Distribution of intestinal parasite species by sex and age group among study participants (*n* = 404).

**Types of intestinal parasites**	**Total infected (prevalence in %)**	**Burden of IPI in sex**		**Burden of IPI age (in years)**		
		**Male (%)**	**Female (%)**	**5–14 (%)**	**15–24 (%)**	**> 25 (%)**
*Prevalence of infection*						
*E. histolytic*	68 (16.83)	31 (45.5)	37 (54.41)	22 (32.35)	26 (38.24)	20 (29.41)
*G. lamblia*	40 (9.90)	16 (40.0)	24 (60.0)	16 (40.0)	14 (35.0)	10 (25.0)
*A. lumbricoides*	33 (8.17)	20 (60.61)	13 (39.39)	9 (27.27)	13 (39.39)	11 (33.33)
Hookworm	31 (7.67)	19 (61.29)	12 (38.71)	13 (41.94)	10 (32.26)	8 (25.81)
*S. mansoni*	17 (4.21)	8 (47.06)	9 (52.94)	5 (29.41)	7 (41.18)	5 (29.41)
*H. nana*	2 (0.50)	2 (100)	0 (0.0)	1 (50.0)	0 (0.0)	1 (50.0)
Overall prevalence	191 (47.28)	96 (50.26)	95 (49.74)	67 (35.08)	70 (36.65)	54 (28.27)
*Double infection (n* = 41)						
*E. histolytica* and *G. lamblia*	6 (14.63)	1 (16.67)	5 (83.33)	2 (33.33)	2 (33.33)	2 (33.33)
*E. histolytica* and *A. lumbricoides*	7 (17.07)	5 (71.43)	2 (28.57)	0 (0.0)	3 (42.86)	4 (57.14)
*E. histolytica* and hookworm	5 (12.2)	4 (80.0)	1 (20.0)	3 (60.0)	1 (20.0)	1 (20.0)
*E. histolytica* and *S. mansoni*	8 (19.51)	3 (37.50)	5 (62.50)	2 (25.0)	5 (62.50)	1 (12.50)
*G. lamblia* and hookworm	7 (17.07)	3 (42.86)	4 (57.14)	4 (57.14)	2 (28.57)	1 (14.29)
*A. lumbricoides* and hookworm	8 (19.51)	6 (75.0)	2 (25.0)	2 (25.0)	2 (25.0)	4 (50.0)
*Triple infection (n* = 2)						
*E. histolytica*, hookworm, and *S. mansoni*	2 (100)	1 (50.0)	1 (50.0)	0 (0.0)	1 (50.0)	1 (50.0)

**Table 3:** Bivariate logistic regression analysis of sociodemographic-related factors and IPIs among study subjects attending at Delgi Primary Hospital.

**Risk factor**	**Category**	**N (%)**	**IPIs**		**COR (95%), ** **p** ** value**
			**Positive (%)**	**Negative (%)**	
Sex	Male	210 (52.0)	72 (34.3)	138 (65.7)	0.865 (0.576, 1.299), 0.484
	Female	194 (48.0)	74 (37.3)	120 (62.4)	1

Age	5–14	151 (37.4)	52 (34.45)	99 (65.6)	0.814 (0.484, 1.370), 0.439
	15–24	151 (37.4)	54 (35.1)	97 (64.5)	2.838 (0.499, 4.409), 0.005⁣^∗^
	>25	102 (25.2)	40 (39.2)	62 (60.8)	1

Residence	Urban	158 (39.1)	53 (32.9)	105 (67.1)	0.807 (0.530, 1.229), 0.317
	Rural	246 (60.9)	93 (37.8)	153 (62.2)	1

Marital status	Single	170 (42.1)	61 (35.3)	109 (64.7)	1.409 (0.674, 2.944), 0.362
	Married	115 (28.5)	45 (39.1)	70 (60.9)	1.661 (0.773, 3.567), 0.193
	Divorced	76 (18.8)	28 (36.8)	48 (63.8)	1.507 (0.668, 3.398), 0.323
	Windowed	43 (10.6)	12 (27.9)	31 (72.1)	1

Educational status	Illiterate	74 (18.3)	30 (37.8)	44 (62.2)	0.969 (0.455, 1.899), 0.928
	Primary	138 (34.8)	51 (37.0)	87 (63.0)	0.934 (0.516, 1.688), 0.820
	Secondary	122 (30.2)	39 (32.0)	83 (68.0)	0.748 (0.405, 1.382), 0.354
	Diploma and above	70 (17.3)	27 (38.6)	43 (61.4)	1

Income level (in ETB)	< 1000	128 (31.7)	44 (34.4)	84 (65.6)	1.893 (0.530, 2.503), 0.004⁣^∗^
	1000–3000	157 (38.9)	58 (36.3)	99 (63.7)	2.672 (0.593, 4.593), 0.014⁣^∗^
	> 3000	119 (29.5)	44 (37.0)	75 (63.0)	1

Family size	≤ 3	83 (20.6)	30 (36.1)	53 (63.9)	1.177 (0.612, 2.265), 0.625
	4–6	127 (31.4)	46 (35.4)	81 (64.6)	1.141 (0.627, 2.079), 0.665
	7–9	117 (29.0)	45 (38.5)	72 (61.5)	1.300 (0.710, 2.381), 0.395
	> 9	77 (29.1)	25 (32.6)	52 (67.5)	1

Occupational status	Govern. employee	58 (14.4)	25 (41.4)	33 (58.6)	1.276 (0.706, 2.305), 0.419
	Merchant	62 (15.3)	21 (33.9)	41 (66.1)	0.926 (0.511, 1.677), 0.800
	Farmer	50 (12.4)	15 (30.0)	35 (70.0)	0.775 (0.398, 1.507), 0.452
	Housewives	15 (3.7)	7 (46.7)	8 (53.3)	1.582 (0.553, 4.526), 0.393
	Student	219 (54.2)	78 (35.6)	141 (64.1)	1

*Note:* 1 = reference value, *N* = total number of cases.

Abbreviation: COR, crude odds ratio.

⁣^∗^Statistically significant at *p* < 0.05.

**Table 4:** Bivariate logistic regression analysis of lifestyle factors associated with IPIs among study subjects attending Delgi Primary Hospital.

**Risk factor**	**Category**	**N** ** (%)**	**IPIs**		**COR (95%), ** **p** ** value**
			**Positive (%)**	**Negative (%)**	
Habit of handwashing before food	Always	138 (34.2)	44 (31.9)	94 (68.1)	1
	Sometimes	83 (20.5)	32 (38.6)	51 (61.4)	1.341 (0.608, 1.768), 0.895
	No	183 (45.3)	69 (37.7)	114 (62.3)	2.866 (0.576–3.302), 0.005⁣^∗^

Habit of shoe wearing	Always	127 (31.4)	53 (41.9)	75 (58.1)	1
	Sometimes	96 (23.8)	36 (37.5)	60 (62.5)	1.353 (0.803, 2.278), 0.256
	No	181 (44.8)	58 (31.5)	123 (68.5)	1.418 (0.938, 2.143), 0.97

Source of drinking water	Tanker water	31 (7.7)	13 (38.7)	18 (61.3)	1.447 (0.651, 3.216), 0.364
	Burrow water	105 (26.0)	44 (41.9)	61 (58.1)	1.653 (0.988, 2.767), 0.056
	Spring water	110 (27.2)	41 (37.3)	69 (62.7)	1.362 (0.814, 2.277), 0.239
	Pipe water	158 (39.1)	48 (30.4)	110 (69.6)	1

Habit of eating unwashed vegetable	Yes	138 (34.2)	54 (39.1)	87 (60.9)	3.046 (0.685, 5.596), 0.016⁣^∗^
	No	266 (65.8)	92 (63.4)	174 (64.5)	1

Habit of eating raw meat	Yes	145 (35.9)	57 (39.3)	88 (60.7)	1.236 (0.808, 1.892), 0.329
	No	259 (64.1)	92 (34.2)	174 (65.8)	1

*Note:* 1 = reference value; *N* = total number of cases.

Abbreviation: COR, crude odds ratio.

⁣^∗^Statistically significant at *p* < 0.05.

**Table 5:** Bivariate logistic regression analysis of personal hygiene related to IPIs among study subjects at Delgi Primary Hospital (2023).

**Variable**	**Category**	**N** ** (%)**	**IPIs**		**COR (95%), ** **p** ** value**
			**Positive (%)**	**Negative (%)**	
Dirty matter under the nail	Yes	166 (41.1)	62 (36.7)	104 (63.3)	2.939 (0.621, 4.418), 0.001⁣^∗^
	No	238 (58.9)	84 (35.3)	154 (64.7)	1
Presence of latrine at home	Yes	233 (57.3)	78 (33.5)	155 (66.5)	1
	No	171 (42.3)	68 (39.2)	103 (60.8)	3.781 (0.518, 5.177), 0.005⁣^∗^
Frequency of latrine use at home	Sometimes	100 (24.8)	32 (32.0)	68 (68.0)	0.746 (0.442, 1.257), 0.271
	Always	133 (33.2)	48 (35.3)	85 (64.7)	1
Habit of handwashing after toilet	Yes	167 (41.3)	62 (36.5)	105 (63.5)	1
	No	237 (58.7)	84 (35.4)	153 (64.6)	2.048 (0.694, 3.583), 0.001⁣^∗^
Frequency of handwashing after toilet	Sometimes	76 (18.8)	28 (36.8)	48 (63.2)	1.075 (0.628, 1.840), 0.012⁣^∗^
	Always	91 (22.8)	34 (37.0)	57 (63.0)	1

*Note:* 1 = reference value; *N* = total number of cases.

Abbreviation: COR, crude odds ratio.

⁣^∗^Statistically significant at *p* < 0.05.

**Table 6:** Multivariate logistic regression analysis of selected risk factors associated with IPIs among study subjects attending Delgi Primary Hospital (2023).

**Risk factor**	**Category**	**N** ** (%)**	**IPIs**		**AOR (95%), ** **p** ** value**
			**Positive (%)**	**Negative (%)**	
Handwashing before food	Yes	221 (54.4)	77 (34.4)	144 (65.6)	1
	No	183 (45.3)	69 (37.7)	114 (62.3)	1.157 (0.764, 1.752), 0.004⁣^∗^

Age group	5–14	151 (37.4)	52 (34.4)	99 (65.5)	1.835 (0.491, 2.419), 0.001⁣^∗^
	15–24	151 (37.4)	54 (35.1)	97 (64.5)	1.866 (0.511, 2.468), 0.502
	>25	102 (25.2)	40 (39.2)	62 (60.8)	1

Income level (in ETB)	<1000	128 (31.7)	44 (34.4)	84 (65.6)	1.046 (0.803, 1.362), 0.012⁣^∗^
	1000–3000	157 (38.9)	58 (36.3)	99 (63.7)	2.157 (0.764, 3.752), 0.014⁣^∗^
	>30000	119 (29.5)	44 (37.0)	75 (63.0)	1

Habit of handwashing after toilet	Yes	167 (41.3)	62 (36.5)	105 (63.5)	3.941 (0.619, 5.432), 0.001⁣^∗^
	No	237 (58.7)	84 (35.4)	153 (64.6)	1

Habit of eating unwashed vegetable	Yes	138 (34.2)	54 (36.6)	84 (63.4)	2.987 (0.642, 4.517), 0.013⁣^∗^
	No	266 (65.8)	92 (63.4)	174 (64.5)	1

Dirty matter under the nail	Yes	166 (41.1)	62 (36.7)	104 (63.3)	3.934 (0.615, 5.417), 0.001⁣^∗^
	No	238 (58.9)	84 (35.3)	154 (64.7)	1

Presence of latrine at home	Yes	233 (57.3)	78 (33.5)	155 (66.5)	1
	No	171 (42.3)	68 (39.2)	103 (60.8)	1.270 (0.27, 1.39), 0.002⁣^∗^

*Note:* 1 = reference value; *N* = total.

Abbreviation: AOR, adjusted odds ratio (multivariate regression model).

⁣^∗^Statistically significant at *p* < 0.05.

## Data Availability

The entire raw data of this study is available in the hands of the authors and will be submitted for reasonable requests.
